# Inhibition of IL-17A in Tumor Microenvironment Augments Cytotoxicity of Tumor-Infiltrating Lymphocytes in Tumor-Bearing Mice

**DOI:** 10.1371/journal.pone.0053131

**Published:** 2013-01-25

**Authors:** Keiji Hayata, Makoto Iwahashi, Toshiyasu Ojima, Masahiro Katsuda, Takeshi Iida, Mikihito Nakamori, Kentaro Ueda, Masaki Nakamura, Motoki Miyazawa, Toshiaki Tsuji, Hiroki Yamaue

**Affiliations:** Second Department of Surgery, School of Medicine, Wakayama Medical University, Wakayama, Japan; Shanghai Jiao Tong University School of Medicine, China

## Abstract

It remains controversial whether IL-17A promotes or inhibits cancer progression. We hypothesized that IL-17A that is locally produced in the tumor microenvironment has an important role in angiogenesis and tumor immunity. We investigated the effect of inhibiting IL-17A at tumor sites on tumor growth and on local and systemic anti-tumor immunity. MC38 or B16 cells were inoculated subcutaneously into mice, and intratumoral injection of an adenovirus vector expressing siRNA against the mouse IL-17A gene (Ad-si-IL-17) significantly inhibited tumor growth in both tumor models compared with control mice. Inhibition of IL-17A at tumor sites significantly suppressed CD31, MMP9, and VEGF expression in tumor tissue. The cytotoxic activity of CD8^+^ T cells from tumor-infiltrating lymphocytes in mice treated with Ad-si-IL-17 was significantly higher than in control mice; however, CD8^+^ T cells from splenocytes had similar activity levels. Suppression of IL-17A at tumor sites led to a Th1-dominant environment, and moreover, eliminated myeloid-derived suppressor cells and regulatory T cells at tumor sites but not in splenocytes. In conclusion, blockade of IL-17A at tumor sites helped suppress tumor growth by inhibiting angiogenesis as well as cytotoxic T lymphocytes activation at tumor sites.

## Introduction

The relationship between cancer and inflammation has long been discussed, and chronic inflammation is reportedly associated with tumor growth via angiogenesis and tumor immunity [1], [2], [3]. IL-17A is a key cytokine in some disorders related to chronic inflammation, including allergy and autoimmune diseases such as rheumatoid arthritis and inflammatory bowel disease [4], [5]. IL-17A increases the production of IL-6, IL-8, TNF, and MMP in macrophages, fibroblasts, and endothelial cells and is therefore considered a proinflammatory cytokine [6].

The relationship between IL-17A and tumors was first reported by Tartour et al in 1999 [7], who showed that IL-17A worked as a tumor growth factor in nude mice, although its mechanism remained unclear. Since then, the influence of IL-17A on tumor development has been studied. In humans, IL-17A expression has been reported in several tumor types, including ovarian cancer, prostate cancer, colon cancer, non–small cell lung cancer, hepatocellular carcinoma, gastric cancer and esophageal cancer [8], [9], [10], [11], [12], [13], [14]. In humans, most of the studies support IL-17A contributing to tumor development. Recently, however, it has been reported that the levels of Th17 cells and the levels of IL-17A in ascites were reduced in more advanced diseases and positively predicted patient outcome in human ovarian cancer because Th17 cells contributed to anti-tumor immunity [15].

In mice, overexpression of IL-17A by gene transduction into tumor cells promoted tumor growth through angiogenesis [16], [17], but seemingly in contrast, IL-17A also suppressed tumor growth via a T-cell dependent mechanism [18], [19]. Subsequently, IL-17^−/−^ mice have been used to determine the endogenous IL-17A functions with regard to tumor progression. One report using B16 melanoma cell lines showed that IL-17A promoted tumor growth via angiogenesis and induced IL-6 production, which in turn activated oncogenic Stat-3, up-regulating prosurvival and proangiogenic genes [20]. However, another report using MC38 colon cancer cell lines showed that IL-17A inhibited tumor growth through antitumor immunity [21]. Consequently, even with the use of knockout mice, it remains controversial whether IL-17A promotes or inhibits cancer progression. However, the role of local IL-17A is not fully understood in the previous studies. We speculated that the contradictory IL-17A effect on tumor growth in previous studies might be due to IL-17A function being different locally versus systemically. We hypothesized that IL-17A produced locally in tumor microenvironment might have an important role on tumor growth via angiogenesis and tumor immunity, thereby tumor development might be suppressed by inhibiting IL-17A at tumor local sites but not systemically. To test this hypothesis, we inhibited IL-17A expression at tumor sites by intratumoral injection of an adenovirus vector expressing IL-17A siRNA (Ad-si-IL-17) in WT mice and examined the effects on tumor progression. In addition, we investigated the mechanism of IL-17A inhibition at tumor sites with regard to tumor growth, especially in terms of local antitumor immunity involving tumor-infiltrating lymphocytes (TILs). We determined the blockade of IL-17A at tumor sites could suppress tumor growth by inhibiting angiogenesis as well as cytotoxic T lymphocytes (CTL) activation at tumor sites.

## Materials and Methods

### Ethics Statement

This study was carried out in strict accordance with the recommendations in the Guide for the Care and Use of Laboratory Animals of the National Institutes of Health. The protocol was approved by the Institutional Animal Care and Use Committees of the School of Medicine, Wakayama Medical University (Permit Number: 440). Murine melanoma cell line B16 and human embryonic kidney cell line 293 were purchased from Japanese Collection of Research Bioresourses (JCRB, Osaka, Japan), and murine chemically induced colon carcinoma cell line MC38 was obtained from Dr. F. James Primus [22].

### Mice and cell lines

Female 6- to 8-wk-old C57BL/6 mice (CLEA Japan, Tokyo, Japan) were used for the experiment. The murine chemically induced colon carcinoma cell line MC38 and human embryonic kidney cell line 293 were maintained in DMEM (Nissui, Tokyo, Japan) supplemented with 10% FBS, 2 mM L-glutamine, 100 U/ml penicillin, and 100 µg/ml streptomycin (Invitrogen, Carlsbad, CA, USA). Murine melanoma cell line B16 was grown in MEM (Invitrogen) supplemented with 10% FBS, 100 U/ml penicillin, and 100 µg/ml streptomycin.

### Construction of the recombinant adenovirus vector

The murine IL-17A expression vector, cloned into pBApo-CMV-Neo vector (Takara, Otsu, Japan), was constructed and two murine IL-17A siRNA expression vectors, cloned into pcPURmU6i vector, were constructed. IL-17A and IL-17A siRNA plasmids cotransfected 293 cells, and we selected the IL-17A siRNA plasmid that could force lower IL-17A expression (data not shown), the sequences was as follows: 5′-GTTTAGGTTAACTTCAAGGTCTTACGTGTGCTGTCCGTAAGACTTTGAGGTTGAGGTTGACCTTTTT-3′. The IL-17A siRNA fragment was excised and ligated into cosmid vector pAxcwit2 (Takara) to yield Ad-si-IL-17. The recombinant Ad-si-IL-17 was generated by the COS-TPC method as previously described [22] and was used in vivo after purification with Vivapure AdenoPACK (Sartorius, Goettingen, Germany).

### Design of in vivo experiments

C57BL/6 mice were inoculated subcutaneously in the right flank with 5×10^5^ MC38 cells (n = 7/group), and at 5, 8, and 11 d, PBS, Ad-SNC, or Ad-si-IL-17 with 1×10^9^ PFU was injected intratumorally. In the same way B16 tumor models were inoculated with 1×10^6^ cells and adenovirus vectors were injected on days 7, 10, and 13. All mice were killed when the long diameter of the tumor reached 2 cm. Tumor size was estimated using the following formula: (short diameter)^2^ × long diameter ×0.52.

### Measurement of cytokine and chemokine content in tumor tissues and in vitro

We prepared tumor homogenates as described previously [23], [24], [25]. In brief, tumor tissues were homogenized in cold PBS, and then sonicated for 10 min. Homogenates were centrifuged at 15,000 rpm for 10 min at 4°C, and the total protein concentrations in supernatants were measured using a BCA protein assay kit (Pierce Biotechnology). The IL-17A, vascular endothelial growth factor (VEGF) and CCL2 content in supernatants were determined by ELISA (R&D Systems, Minneapolis, USA). For IL-6 assay, content in supernatants was measured using cytometric bead array system (CBA) (BD Biosciences), following the manufacturer's manual. For the VEGF and IL-17A levels produced by MC38 and B16 in vitro, 2×10^5^ cells were cultured for 48 h in 6-well plate and the supernatants were measured using ELISA kit.

### Preparation of splenocytes and TILs

Spleens were filtered through a 70-µm nylon mesh, and live splenocytes were collected by 100% Ficoll-PREMIUM (GE Healthcare, Piscataway, NJ, USA) gradient centrifugation. Tumor tissues were dissected and digested in complete RPMI1640 medium supplemented with 10% FBS, 2 mM L-glutamine, 50 µM 2-ME (Wako), 100 U/ml penicillin and 100 µg/ml streptomycin, 1 mg/ml collagenase, and 0.2 mg/ml hyaluronidase (Sigma) at 37°C for 30 min; 0.5 mg/ml DNaseI (Sigma) was then added and the suspension was left at 37°C for another 15 min. The tumor suspension was next filtered through a 70-µm nylon mesh and live lymphocytes were collected by 80%/100% Ficoll gradient centrifugation. For T-cell sorting, CD5^+^ cells were isolated from the splenocytes or TILs using autoMACS (Miltenyi Biotec, Bergisch Gladbach, Germany).

### Cytotoxicity assay

Spleens and tumor tissues were removed at 14 d after MC38 cell inoculation, and then in vivo primed splenocytes or TILs were cocultured (4×10^6^/ml) with irradiated (7,500 rad) MC38 cells (4×10^4^/ml) in complete RPMI1640 medium containing recombinant IL-2 at 50 IU/ml. TILs were pooled from four to five mice. After 72 h of coculture, CD8^+^ T cells were isolated from in vitro restimulated splenocytes or TILs using autoMACS. Cytotoxicity was tested using a 4 h ^51^Cr-release assay, as described previously [22].

### Flow cytometric analysis

For analysis of MDSCs, splenocytes or TILs were stained with PerCP -Cy5.5–conjugated anti-CD45, FITC-conjugated anti-CD11b, and PE-conjugated anti-Gr-1 mAb (BD) for 30 min on ice. For analysis of Treg cells, splenocytes or TILs stained with PerCP -Cy5.5–conjugated anti-CD3, and FITC-conjugated anti-CD4 mAb (BD) for 30 min on ice. For intracellular staining, after fixation and permeabilization using Foxp3 Staining Buffer Set (eBioscience), the cells were stained with PE-conjugated anti-FOXP3 mAb (eBioscience) for 30 min at 4°C. For analysis of Th1/Th2/Th17 cells, splenocytes or TILs were stimulated with PMA (10 ng/ml) and ionomycin (500 ng/ml) (Sigma) for 5 h in the presence of GolgiPlug (BD). Then, the cells were harvested and stained with PerCP -Cy5.5–conjugated anti-CD4 mAb (BD) for 30 min on ice. For intracellular staining, after fixation and permeabilization using BD Cytofix/Cytoperm (BD), the cells were stained with FITC-conjugated anti-IFN-γ, PE-conjugated anti-IL-4 mAb, PE-conjugated anti-IL-17 mAb (BD) for 30 min on 4°C. After washing, the cells were analyzed by FACSCalibur, using CELLQuest (BD Biosciences).

### Immunohistochemistry and quantitative microscopy

For immunohistochemical staining of CD31 specific for endothelial tissue, 10-µm sections were prepared from frozen tumor tissues. For immunohistochemical staining of MMP9, 4-µm sections were prepared from paraffin-embedded blocks derived from tumor tissues. The primary antibodies, rat anti-CD31 mAb (1∶100, BD) and rabbit anti-MMP9 Ab (1∶100, R&D), were incubated overnight at 4°C. The immunocomplex was visualized by a polymer envision method, histofine simple stain (Nichirei, Tokyo, Japan). Vessel counts were assessed according to the criteria of Weidner et al [26]. Vessels in five high-power fields (×200 magnification) were counted. Positive cells were quantified by an image-processing application (Win ROOF, version 5.5; Mitani).

### Statistical analysis

**Figure 1:**
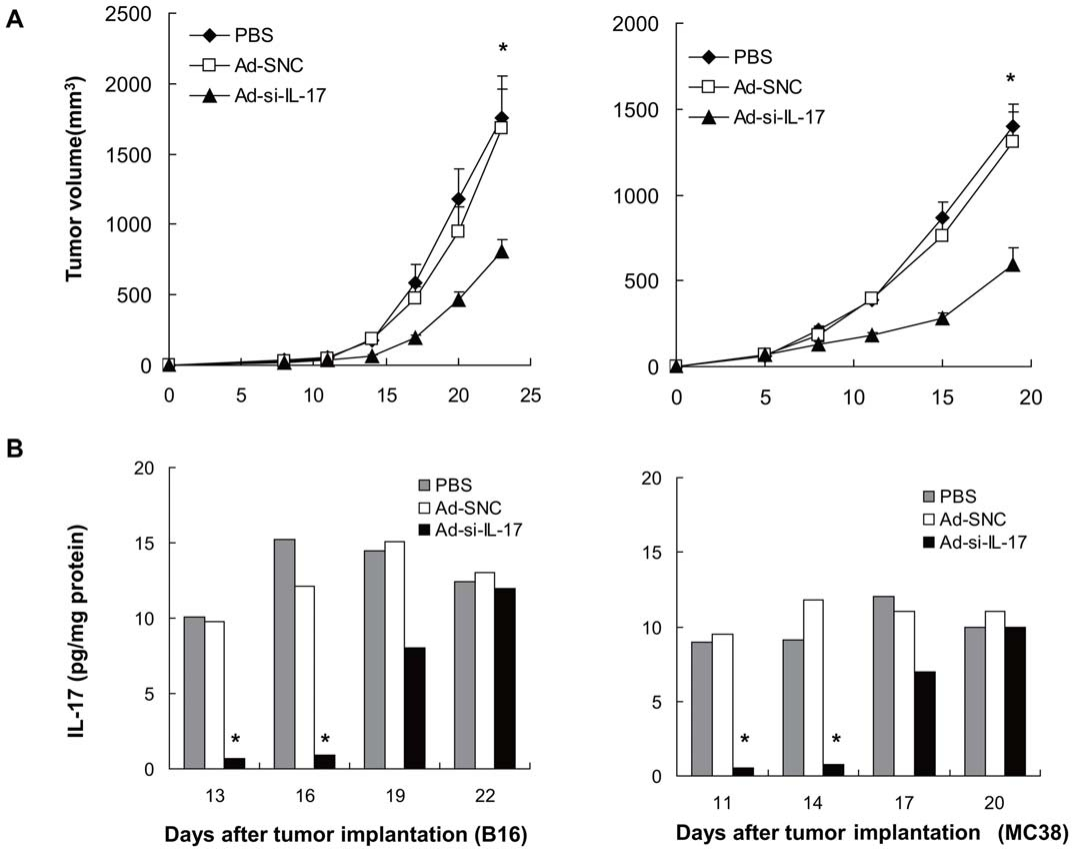
Inhibition of IL-17A at tumor sites suppresses tumor growth. (A, left panel) 1×10^6^ B16 tumor cells were injected subcutaneously into C57BL/6 mice, and PBS, Ad-SNC, or Ad-si-IL-17 with 1×10^9^ PFU was injected intratumorally at 7, 10, and 13 d. Data represent means ± SE (*n* = 7 mice per group of two independent experiments). *P<0.05, one-way ANOVA. (A, right panel) 5×10^5^ MC38 tumor cells were injected subcutaneously into mice, in the same way adenovirus vectors were injected at 5, 8, and 11 d. Data represent means ± SE (*n* = 7 mice of two independent experiments). *P<0.05, one-way ANOVA. (B).Levels of IL-17A protein were measured by ELISA in tumor lysates from mice after having been treated with PBS, Ad-SNC, or Ad-si-IL-17. In B16 and MC38 tumor tissues, Ad-si-IL-17 treatment showed lower levels of IL-17A compared with PBS or Ad-SNC treatment. Data are representative of two independent experiments. Data are presented as means ± SE (*n* = 4). *P<0.05, Student's *t* test.

The following statistical analyses were used. For data in Figures 1(A), we used the one-way ANOVA. A Student's *t* test or a Mann-Whitney test was used to assess the statistical significance of the differences between experimental and control groups. All statistical analyses were performed with StatView 5.0 (Abacus Concepts, Inc, Berkeley, California) statistical software program. A value of *P*<0.05 was considered statistically significant.

## Results

### Inhibition of IL-17A expression at tumor sites suppresses tumor growth

**Figure 2:**
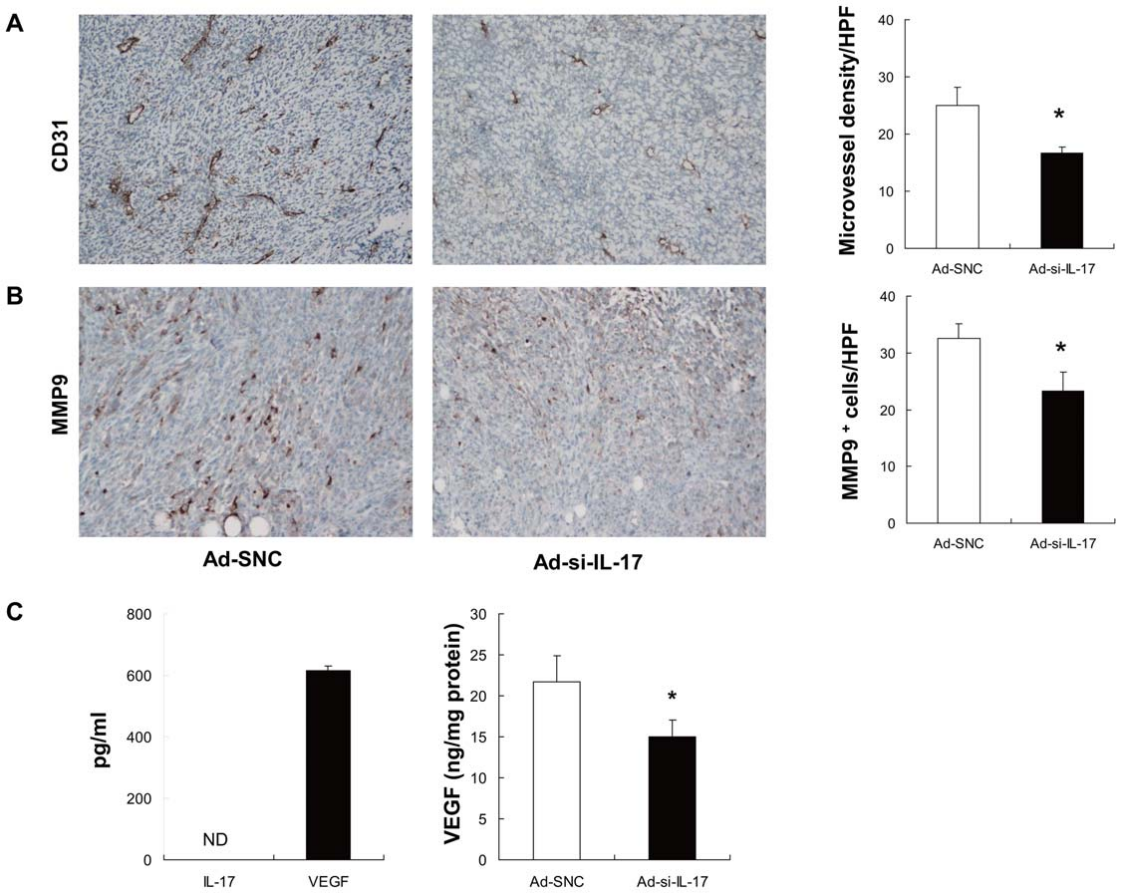
Inhibition of IL-17A at tumor sites decreases the intratumoral microvessel density. (A) The endothelial maker CD31-stained sections of tumor tissue are shown in Ad-SNC and Ad-si-IL-17 treatment models, Ad-si-IL-17 treatment decreased the intratumoral microvessel density compared with Ad-SNC. Vessels in five high-power fields (×200 magnification) were counted. Positive cells were quantified by an image-processing application. Data are representative of two independent experiments. Data are presented as means ± SE (*n* = 5). *P<0.05, Student's *t* test. (B) Intratumoral MMP-9 expression was decreased by the inhibition of IL-17. MMP-9 stained sections of tumor tissue were shown in Ad-SNC and Ad-si-IL-17 treatment models. Ad-si-IL-17 treatment decreased the intratumoral MMP-9 density compared with Ad-SNC. MMP-9 in five high-power fields (×200 magnification) was counted. Positive cells were quantified by an image-processing application. Data are representative of two independent experiments. Data are presented as means ± SE (*n* = 5). *P<0.05, Student's *t* test. (C) VEGF and IL-17A levels produced by MC38 cells were measured by ELISA. MC38 cells did not secretion IL-17A protein and produce VEGF protein. Levels of VEGF in vivo were measured by ELISA in tumor lysates from mice at 14 d after having been treated with Ad-SNC, or Ad-si-IL-17. In tumor tissues, Ad-si-IL-17 treatment showed lower levels of VEGF compared with Ad-SNC treatment. Data are representative of two independent experiments. Data are presented as means ± SE (*n* = 4). *P<0.05, Student's *t* test. ND: not detected.

We first examined how inhibiting locally expressed IL-17A affected tumor growth by using a B16 subcutaneous tumor model. Intratumoral injection of Ad-si-IL-17 significantly suppressed tumor growth compared with an adenovirus vector expressing scramble negative siRNA (Ad-SNC) or PBS (*P*<0.05, Fig. 1A, left panel). Next, we examined the effect of inhibiting locally expressed IL-17 on tumor growth by using MC38 subcutaneous tumor models. Intratumoral injection of Ad-si-IL-17 also significantly suppressed tumor growth compared with Ad-SNC or PBS (*P*<0.01, Fig. 1A, right panel). We confirmed that intratumoral administration of Ad-si-IL-17 significantly suppressed endogenous IL-17A protein expression in vivo (*P*<0.05, Fig. 1B). There was no IL-17A secretion from MC38 and B16 in vitro (Fig. 2C and Fig. S1). These data suggested that endogenously expressed IL-17A at tumor local sites may promote tumor growth regardless of the tumor cell line.

### Inhibition of IL-17 at tumor sites decreases intratumoral microvessel density and MMP9 expression

Next, to investigate the mechanisms of tumor growth suppression by IL-17A inhibition, we performed immunohistochemistry of tumor tissues with regard to angiogenesis. We performed immunohistochemical staining with an anti-CD31 antibody specific for endothelial cells and anti-MMP9 antibody. MMP9 has been mediator of tumor angiogenesis [27]. The number of vascular endothelial cells was significantly lower in tumor tissues treated with Ad-si-IL-17 than with Ad-SNC (*P*<0.05, Fig. 2A). MMP9 expression in tumor tissues also decreased when treated with Ad-si-IL-17 compared with Ad-SNC (*P*<0.05, Fig. 2B). In addition, VEGF was down regulated by inhibiting of IL-17A in tumor tissues (*P*<0.05, Fig. 2C and Fig. S1). These finding showed that blockade of IL-17A at tumor sites might inhibit tumor angiogenesis.

### Inhibition of IL-17A at tumor sites improves CTL activation in tumor microenvironment but not systemically

**Figure 3:**
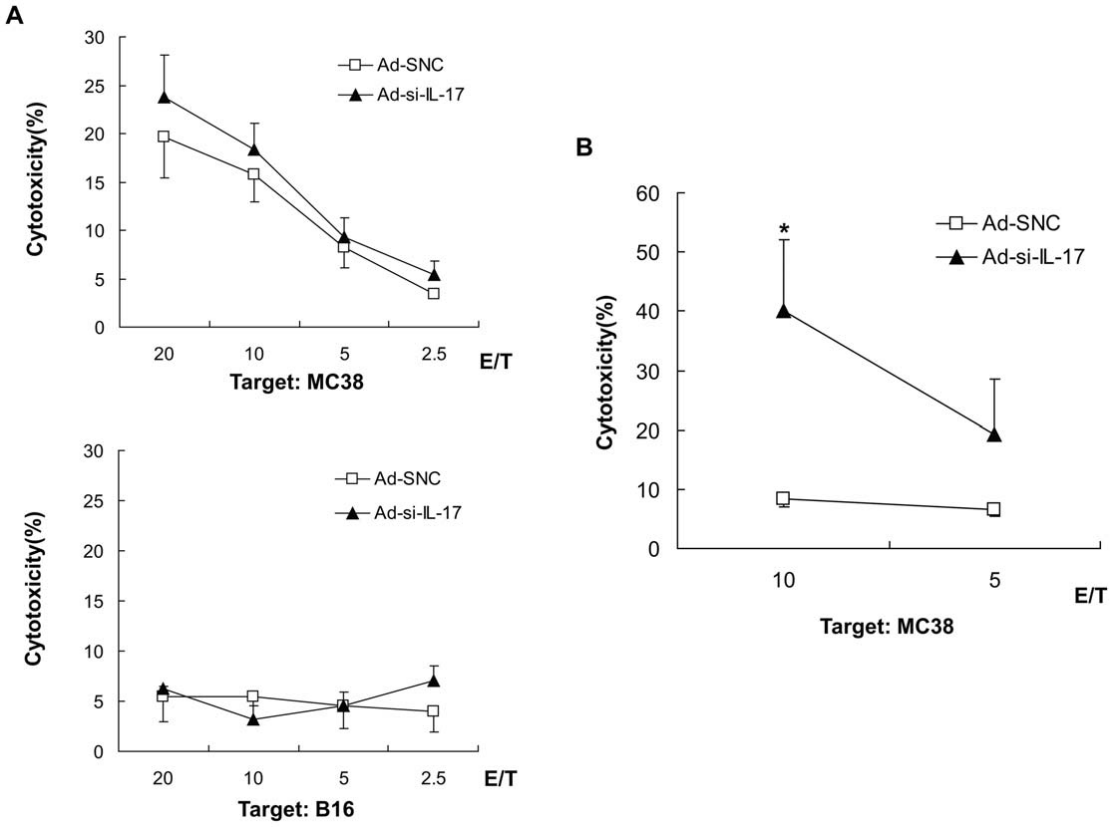
Inhibition of IL-17A in tumor sites promotes CTLs activation, especially in the tumor microenvironment. The cytotoxicity assay used CD8^+^ T cells from splenocytes or TILs in MC38 subcutaneous tumor treated Ad-si-IL-17 and Ad-SNC. (A) The cytotoxic activities against MC38 cells of CD8^+^ T cells from splenocytes in mice treated with intratumoral injection of Ad-si-IL-17 were almost the same as those in control mice (*n* = 5). The cytotoxic activities against B16 cells were less than 10% in both groups (*n* = 5). (B) The cytotoxic activities against MC38 cells in CD8^+^ T cells from TILs in mice treated with intratumoral injection of Ad-si-IL-17 were significantly higher than those with Ad-SNC (*n* = 3). Data are representative of two independent experiments. Data are presented as means ± SE. *P<0.05, Mann-Whitney test.

To investigate the effect of the inhibition of IL-17A in tumor tissue on systemic and local anti-tumor immunity, we assessed the cytotoxic activity of CD8^+^ T cells from splenocytes or TILs in the MC38 subcutaneous model. The systemic CTL responses in mice treated with Ad-si-IL-17 treatment were first compared to those in control mice. The cytotoxic activities against MC38 cells of CD8^+^ T cells from splenocytes in mice treated with intratumoral injection of Ad-si-IL-17 were almost the same as those from mice treated with Ad-SNC (Fig. 3A, upper panel), and the cytotoxic activities against B16 cells as control targets were less than 10% in both groups (Fig. 3A, lower panel). However, the CTL responses in TILs from tumor tissues in mice treated with Ad-si-IL-17 were compared to those in control mice and found to differ. The cytotoxic activities against MC38 cells of CD8^+^ T cells from TILs in mice treated with intratumoral injection of Ad-si-IL-17 were significantly higher than those in mice treated with Ad-SNC (*P*<0.05, Fig. 3B). In the B16 subcutaneous model, the cytotoxicity of CD8^+^ T cells from TILs also significantly increased by IL-17A inhibiting in tumors compared to controls, but cytotoxicity of CD8^+^ T cells from splenocytes was same levels compared to controls (Fig. S2). These data suggested that inhibition of IL-17A in tumor tissues caused CTL activation in TILs but not in spleen cells.

### Inhibition of IL-17A at tumor sites increases Th1 cells in tumor microenvironment

**Figure 4:**
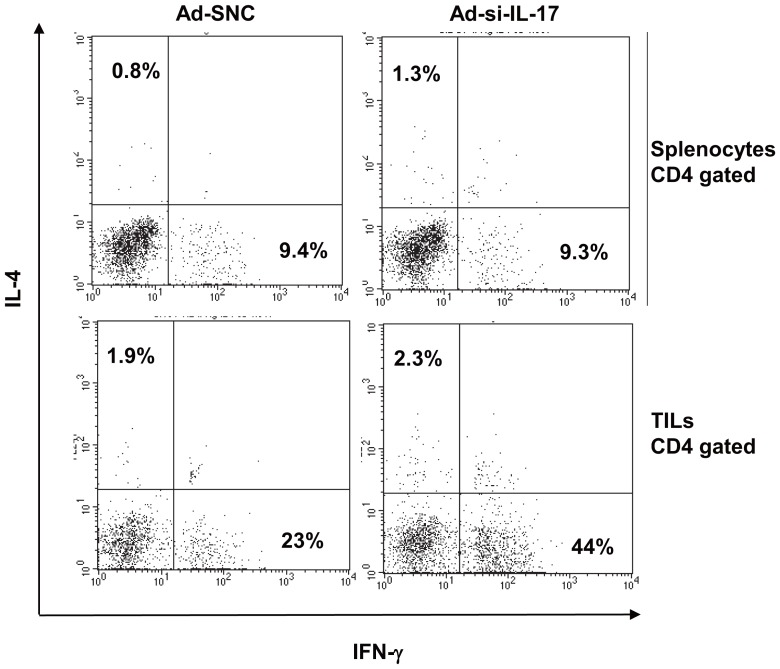
Inhibition of IL-17A in tumor sites increases Th1 cells in tumor microenvironment. Splenocytes and TILs were collected from spleens or tumor tissues of MC38 subcutaneous tumor model. Th1/Th2 cells were detected by intracellular staining assay of anti-IFN-γ mAb and anti-IL-4 mAb. Representative flow cytometry analysis profiles gated on anti-CD4 mAb. Ad-si-IL-17 treatment increased Th1 phenotype of TILs compared to control, but that of splenocytes was similar level in both treatments. Th2 phenotype was similar ratio of TILs and splenocytes in both treatment. Data are representative of two independent experiments (*n* = 5).

To examine why CTL activity, especially in the tumor microenvironment, was enhanced by inhibiting IL-17A in tumor tissues, we examined the Th1/Th2 phenotype of T lymphocytes of spleen cells and TILs in the MC38 subcutaneous model. Populations of both IL-4^+^ CD4^+^ T cells and IFN-γ^+^ CD4^+^ T cells in splenocytes were at similar levels in Ad-si-IL-17–injected mice and control mice (Fig. 4, Table 1). On the other hand, the levels of IFN-γ^+^ CD4^+^ T cells in TILs were significantly higher in Ad-si-IL-17–injected mice than in control mice; however, IL-4^+^ CD4^+^ T cells were at similar levels in both types of mice (*P*<0.01, Fig. 4, Table 1). In the B16 subcutaneous model, the percentage of IFN-γ^+^ CD4^+^ T cells in TILs with Ad-si-IL17 was also more increased compared to control mice. There were no differences the population of Th2 cells in TILs and Th1/Th2 cells in splenocytes with Ad-si-IL-17 compared to controls (Fig. S3, Table S1). There was no significant difference in Th1/Th2 cytokines, such as IL-2, IL-4, IL-10, TNF-α, IFN-γ, levels of tumor lysate in both types of mice (data not shown). These findings indicated blockade of IL-17A at tumor sites might shift to Th1 dominance in tumor microenvironment.

**Table 1:** The levels of each immune subset in splenocytes and TILs (MC38).

	Splenocytes		TILs	
Cells	Ad-SNC	Ad-si-IL-17	Ad-SNC	Ad-si-IL-17
IFN-γ^+^ CD4^+^	8.0±0.8	8.0±0.5	15±2	33±5*
IL-4^+^ CD4^+^	1.0±0.2	1.1±0.2	4.4±0.8	6.1±1
Foxp3^+^ CD4^+^	13±1	13±1	39±2	29±2*
Gr1^+^ CD11b^+^ CD45^+^	6.0±0.2	6.4±0.5	31±0.7	26±1*

Spleens were filtered through mesh, and live splenocytes were collected by 100% Ficoll gradient centrifugation. Tumor tissues were dissected and digested, and filtered through mesh, and TILs were collected by 80%/100% Ficoll gradient centrifugation. Splenocytes or TILs were stained with anti-CD4, anti-IFN-γ, anti-IL-4, anti-Foxp3, anti-Gr-1, anti-CD11b and anti-CD45 mAb. Data are representative of two independent experiments. Values represent the mean percentage ± SE in CD4^+^ or CD45^+^ population. (*n* = 5 per group, *P<0.05 (Student's *t* test), compared with Ad-SNC).

### Myeloid-derived suppuressor cells (MDSCs) and regulatory T (Treg) cells are eliminated by inhibiting IL-17A in tumor microenvironment

**Figure 5:**
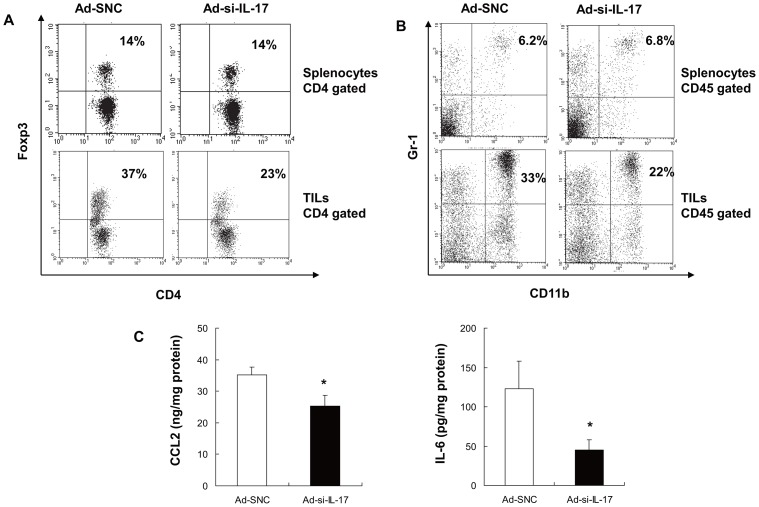
Inhibition of IL-17A at tumor sites reduces MDSCs and Treg cells in the tumor microenvironment. (A) Splenocytes and TILs were collected from spleens or tumor tissues of MC38 subcutaneous tumor model. Treg cells were detected by intracellular staining assay of anti-Foxp3 mAb. Representive flow cytometry analysis profiles gated on anti-CD3 and anti-CD4 mAb. Ad-si-IL-17 treatment decreased the ratio of Treg cells in TILs, but those in splenocytes were similar in both treatments. Data are representative of two independent experiments (*n* = 5). (B) Splenocytes and TILs were collected from spleens or tumor tissues of the MC38 subcutaneous tumor model. MDSCs cells were detected by double-staining of anti-CD11b mAb and anti-Gr1 mAb. Representive flow cytometry analysis profiles gated on anti-CD45 mAb. Ad-si-IL-17 treatment decreased the ratio of MDSCs in TILs, but those in splenocytes were at similar levels in both treatments. Data are representative of two independent experiments (*n* = 5). (C) Levels of IL-6 and CCL2 protein were measured in tumor lysates from mice at 14 d after having been treated with Ad-SNC, or Ad-si-IL-17. IL-6 and CCL2 were significantly decreased in tumors from Ad-si-IL-17–injected mice compared with control mice. Data are presented as means ± SE (*n* = 5). *P<0.05, Student's *t* test.

Next, to investigate the mechanism by which Th1 cells and CTLs were activated though inhibition of IL-17A in the tumor microenvironment, we assessed immunosuppressor cells, such as MDSCs and Treg cells, in splenocytes or TILs. The populations of both levels of Foxp3^+^ CD4^+^ T cells and Gr1^+^ CD11b^+^ cells in splenocytes were at similar levels in Ad-si-IL-17-injected mice and control mice (Fig. 5A and 5B, Table 1, Fig. S4, Table S1). On the other hand, the populations of both Gr1^+^ CD11b^+^ cells and Foxp3^+^ CD4^+^ T cells in the MC38 tumors were significantly lower in Ad-si-IL-17–injected mice compared with control mice (*P*<0.05, Fig. 5A and 5B, Table 1). In the B16 subcutaneous model, the percentage of Gr1^+^ CD11b^+^ cells in TILs with Ad-si-IL17 were also more decreased compared to control mice, but there were no differences in the percentage of Foxp3^+^ CD4^+^ T cells in TILs with Ad-si-IL17 compared to controls (Fig. S4, Table S1). These results suggested that both MDSCs and Treg cells in tumor microenvironment may be eliminated by inhibiting IL-17A at tumor sites. Next, we measured the IL-6 and CCL2 in tumors associated with proliferation and migration of Gr1^+^ CD11b^+^ cells [28], [29]. IL-6 and CCL2 were significantly decreased in tumors from Ad-si-IL-17–injected mice compared with control mice (Fig. 5C, Fig. S4). The lower concentration of IL-6 and CCL2 might explain the reduction of Gr1^+^ CD11b^+^ cells in tumors from Ad-si-IL-17–injected mice.

## Discussion

In the present study, we clearly showed that inhibition of naturally expressed IL-17A at tumor sites suppressed tumor growth. We selected two different subcutaneous tumor models in which controversial results have been reported using IL-17^−/−^ mice. One was the MC38 subcutaneous tumor model, which promoted tumor progression using IL-17^−/−^ mice [21], and the other was the B16 subcutaneous model, which suppressed tumor growth [20]. However, these studies using IL17^−/−^ mice had several limitations. IL-17^−/−^ mice might have a potential defect of innate immunity function because IFN-γ^+^ NK cells and IFN-γ^+^ T cell in LN and spleen were also moderately reduced in IL-17^−/−^ mice without tumors compared with WT mice [21]. In the studies using IL-17^−/−^ mice, the role of systemic IL-17A could be evaluated on tumor progression and, however, the role of local IL-17A on tumor progression could not to be understood. Therefore, we planned to inhibit only local IL-17A at tumor sites by intratumoral injection of Ad-si-IL-17 and examined the role of IL-17A in tumor microenvironment on tumor growth. We demonstrated that blockade of local IL-17A led to not only inhibiting angiogenesis but also CTL activation in TILs. These results supported our hypothesis that IL-17A produced locally in the tumor microenvironment might have an important role on tumor growth.

IL-17A has tumor-promoting effects, especially in the context of inflammation and angiogenesis [16], [17], [30], [31], [32]. In vivo the microvessel density in tumor tissues is increased by overexpression of IL-17A at tumor sites and decreased in IL-17^−/−^ mice [16], [32]. IL-17A up-regulates production of a variety of proangiogenic factors in vitro, such as VEGF, prostaglandin E1 and E2, and macrophage inflammatory protein-2, by fibroblasts as well as tumor cells [16]. Our results were consistent with those from previous reports, and revealed that the inhibition of IL-17A expressed in tumor tissue significantly suppressed the microvessel density compared with a control model and was accompanied by lower expression of MMP9 and VEGF.

From the standpoint of tumor immunity, it remains controversial whether IL-17A behaves as an antitumor or protumor factor. The overexpression of IL-17A at tumor sites suppressed tumor progression through the enhanced tumor-specific CTL response in immunocompetent mice [18], [19]. However, overexpression models may be artificial, because these cells could produce much higher levels of IL-17A than those in a general tumor microenvironment. Therefore, these models are not appropriate for evaluating the function of IL-17A expressed in a tumor microenvironment. To assess the role of the endogenous IL-17A, IL-17^−/−^ mice were used in previous studies. One report indicated that IFN-γ^+^ NK cells and IFN-γ^+^ T cells were decreased in the tumor tissue and tumor-draining LN in IL-17^−/−^ mice compared with WT mice, resulting in promotion of tumor growth and lung metastasis [21]. On the other hand, in another report using IL-17^−/−^ mice, tumor-infiltrating CD8^+^ T cells in IL-17^−/−^ and IL-17R^−/−^ mice were increased and the tumor-infiltrating CD8^+^ T cells from IL-17^−/−^ mice produced more IFN-γ compared with WT mice [20], [33]. Recently, it has reported that IL-17-exposed monocytes suppressed cytotoxic T-cell immunity in vitro [34]. Although these studies seem to suggest that the compete elimination of IL-17A favor the migration of CD8^+^ T cells to tumor sites leading to augmentation of antitumor immunity, the cytotoxic activity of TILs is not fully understood. In the present study, we showed that cytotoxicity of CD8^+^ T cells from TILs was activated by inhibiting IL-17A at tumor sites, and in contrast, the cytotoxicity of CD8^+^ T cells from spleen cells was not affected. To elucidate the mechanism of activation of CTLs, we focused on Th1/Th2 balance and the involvement of immunosuppressor cells.

It is generally known that Th1 cytokines, such as IFN-γ, and Th2 cytokine, such as IL-4, regulate the differentiation and development of Th17 cells; however, the effect of IL-17 on the Th1/Th2 balance has yet to be clarified. In IL-17^−/−^ mice, IL-17A does not influence Th2 [20], [21], but the effect of IL-17A on Th1 remains controversial. One report showed that tumor-infiltrating IFN-γ^+^ CD4^+^ T cells were increased in IL-17^−/−^ mice compared with WT mice [20]; conversely, another report showed that these cells were decreased in IL-17^−/−^ mice [21]. Recently it has been reported that IL-17A directly inhibits Th1 cells because IL-17A binds to its receptor expressed on Th1 cells and specifically inhibits Th1 functions by inhibiting the master transcription factor T-bet [35], [36], [37]. These reports support our results that the inhibition of IL-17A at tumor sites increased Th1 cells in TILs, but did not influence Th1 cells in spleen cells. On the other hand, the relationship between Th17 cells and tumor progression remains to be elucidated. Adaptive transfer of Th17 cell reduced tumor growth because Th17 cells developed into Th1 cells in vivo and produced INF-γ and promoted the activation of CTLs [38], [39]. Namely, Th17 cells have a remarkable plasticity and the transfer of large number of Th17 cells such as cell therapy could show the antitumor effect. However, conversely, Th17 cells that naturally exist in vivo promote tumorigenesis [30]. We examined the population of Th17 cells and Th1 cells in both TILs and splenocytes in subcutaneous tumor model by flow cytometry analysis. The results showed that Th17 cells were decreased in TILs with Ad-si-IL-17, and, in contrast, Th1 cells were increased in TILs with Ad-si- IL-17. On the other hand, there were no differences in the population of Th17 cells and Th1 cells in splenocytes with Ad-si-IL-17 (Fig. S5). However, the role of Th17 cell on tumor growth remains to be clarified because in the present study we focused on the effect of local IL-17A on tumor growth but not on the function of Th17 cells.

The relationship between IL-17A and immunosuppressor cells such as MDSCs or Treg cells also remains unclear. MDSCs produce nitric oxide and reactive oxygen species and suppress T-cell function through modification of T-cell receptors, inhibition of JAK3 and STAT5, inhibition of MHC class II expression, and induction of T-cell apoptosis [29]. Non-immunological functions of MDSC have also been described, such as the promotion of tumor angiogenesis and metastasis [40]. The expansion and activation of MDSC is influenced by several factor. CCL2 is known to be a potent chemoattractant for MDSCs [29], [41], and IL-6 has an impact on survival and proliferation of MDSCs in tumor microenvironment [28]. IL-6 promotes the differentiation of Th17 cells [42], and IL-17A also amplifies IL-6 production in the tumor [20], [43], [44]. Most recently, it has been reported that IL-17A promotes the infiltration of MDSCs at tumor sites and augments the development and function of MDSCs [33], [45], [46]. In fact, in the present study, inhibiting of IL-17A at tumor sites decreased IL-6 and CCL2 in tumors, and the migration of MDSCs in tumor tissues was obviously reduced. It seemed to be associated with cytotoxic activity of CD8^+^ T cells in TILs. On the other hand, Th17 has been thought to be in a reciprocal relationship with the development of Treg cells, because IL-6 with TGF-β has led to Th17 development, whereas it inhibits Treg development [47]. However, some reports have shown a parallel expansion of both Th17 and Treg responses in tumor microenvironment [37], [48]. Furthermore, IL-17A produced by MDSCs recruits Treg cells at tumor sites via up-regulation of chemokines CCL17 and CCL22 and enhances their suppressor function via up-regulation of CD39 and CD73 [49]. These studies suggest that IL-17A induces MDSCs at tumor sites and also recruits Treg cells to tumor sites, resulting in potent inhibition of T-cell function. In our study, inhibiting of IL-17A at tumor sites decreased tumor- infiltrating Treg cells in MC38 subcutaneous model, but not in B16 subcutaneous model. Because Treg cells tended to more infiltrate in MC38 tumors than in B16 tumors, the inhibitory effect of Treg cells by blocking of IL-17A might was elicited stronger in MC38 model than in B16 model.

Considering our results on tumor immunity, IL-17A at local tumor sites presumably induces MDSCs through IL-6 and CCL2, recruits Treg cells and directly inhibits Th1 functions or indirectly suppresses Th1 functions by MDSCs and Treg cells, finally resulting in anergy and dysfunction of CTLs in tumor sites. Therefore, blocking IL-17A at tumor sites decreased MDSCs and Treg cells and caused the Th1 shift. Subsequently, cytotoxic activity of CTLs in tumor sites was augmented. Improvement of the antitumor immunity resulted in tumor reduction.

In conclusion, blockade of IL-17A at tumor sites suppressed tumor growth. It inhibited angiogenesis in tumor tissues. It also improved the immunological tumor microenvironment by shifting to Th1 dominance and suppressing MDSCs and Treg cells, resulting in CTL activation at tumor sites. The role of local IL-17A might indicate parallel development of inflammation, causing angiogenesis and immunosuppression in tumor microenvironment.

## Supporting Information

Figure S1 **VEGF and IL-17A levels produced by B16 cells were measured by ELISA.** B16 cells did not secretion IL-17A protein and produce VEGF protein. Levels of VEGF in vivo were measured by ELISA in tumor lysates from mice at 16 d after having been treated with Ad-SNC, or Ad-si-IL-17. In tumor tissues, Ad-si-IL-17 treatment showed lower levels of VEGF compared with Ad-SNC treatment. Data are presented as means ± SE (*n* = 4). *P<0.05, Student's *t* test. ND: not detected.
(EPS)

Figure S2 **The cytotoxicity assay used CD8^+^ T cells from splenocytes or TILs in B16 subcutaneous tumor treated Ad-si-IL-17 and Ad-SNC.** (A) The cytotoxic activities against B16 cells of CD8^+^ T cells from splenocytes in mice treated with intratumoral injection of Ad-si-IL-17 were almost the same as those in control mice (*n* = 4). The cytotoxic activities against MC38 cells were less than 2% in both groups (*n* = 4). (B) The cytotoxic activities against B16 cells in CD8^+^ T cells from TILs in mice treated with intratumoral injection of Ad-si-IL-17 were significantly higher than those with Ad-SNC (*n* = 3). Data are presented as means ± SE. *P<0.05, Mann-Whitney test.
(EPS)

Figure S3 **Splenocytes and TILs were collected from spleens or tumor tissues of B16 subcutaneous tumor model (** ***n*** **= 4).** Th1/Th2 cells were detected by intracellular staining assay of anti-IFN-γ mAb and anti-IL-4 mAb. Representative flow cytometry analysis profiles gated on anti-CD4 mAb. Ad-si-IL-17 treatment increased Th1 phenotype of TILs compared to control, but that of splenocytes was similar level in both treatments. Th2 phenotype was similar ratio of TILs and splenocytes in both treatment.
(EPS)

Figure S4 (A) Splenocytes and TILs were collected from spleens or tumor tissues of B16 subcutaneous tumor model (*n* = 4). Treg cells were detected by intracellular staining assay of anti-Foxp3 mAb. Representive flow cytometry analysis profiles gated on anti-CD3 and anti-CD4 mAb. Treg cells were similar ratio of TILs and splenocytes in both treatment. (B) Splenocytes and TILs were collected from spleens or tumor tissues of the B16 subcutaneous tumor model (*n* = 4). MDSCs cells were detected by double-staining of anti-CD11b mAb and anti-Gr1 mAb. Representive flow cytometry analysis profiles gated on anti-CD45 mAb. Ad-si-IL-17 treatment decreased the ratio of MDSCs in TILs, but those in splenocytes were at similar levels in both treatments. (C) Levels of IL-6 and CCL2 protein were measured in tumor lysates from mice at 16 d after having been treated with Ad-SNC, or Ad-si-IL-17. IL-6 and CCL2 were significantly decreased in tumors from Ad-si-IL-17–injected mice compared with control mice. Data are presented as means ± SE (*n* = 4). *P<0.05, Student's *t* test.
(EPS)

Figure S5 **Splenocytes and TILs were collected from spleens or tumor tissues of MC38 subcutaneous tumor model (** ***n*** **= 4).** Th1/Th17 cells were detected by intracellular staining assay of anti-IFN-γ mAb and anti-IL-17 mAb. Representative flow cytometry analysis profiles gated on anti-CD4 mAb. Ad-si-IL-17 treatment increased Th1 phenotype of TILs compared to control, but that of splenocytes was similar level in both treatments. Conversely, Ad-si-IL-17 treatment decreased Th17 phenotype of TILs compared to control, but that of splenocytes was similar level in both treatments.
(EPS)

Table S1 **Spleens were filtered through mesh, and live splenocytes were collected by 100% Ficoll gradient centrifugation.** Tumor tissues were dissected and digested, and filtered through mesh, and TILs were collected by 80%/100% Ficoll gradient centrifugation. Splenocytes or TILs were stained with anti-CD4, anti-IFN-γ, anti-IL-4, anti-Foxp3, anti-Gr-1, anti-CD11b and anti-CD45 mAb. Values represent the mean percentage ± SE in CD4^+^ or CD45^+^ population. (*n* = 4 per group, *P<0.05 (Student's *t* test), compared with Ad-SNC).
(EPS)

## References

[1] Lin WW, Karin M (2007) A cytokine-mediated link between innate immunity, inflammation, and cancer. J Clin Invest 117: 1175–1183. 17476347 10.1172/JCI31537 PMC1857251

[2] Coussens LM, Werb Z (2002) Inflammation and cancer. Nature 420: 860–867. 12490959 10.1038/nature01322 PMC2803035

[3] Mantovani A, Romero P, Palucka AK, Marincola FM (2008) Tumour immunity: effector response to tumour and role of the microenvironment. Lancet 371: 771–783. 18275997 10.1016/S0140-6736(08)60241-X

[4] Fujino S, Andoh A, Bamba S, Ogawa A, Hata K, et al (2003) Increased expression of interleukin 17 in inflammatory bowel disease. Gut 52: 65–70. 12477762 10.1136/gut.52.1.65 PMC1773503

[5] Chabaud M, Durand JM, Buchs N, Fossiez F, Page G, et al (1999) Human interleukin-17: A T cell-derived proinflammatory cytokine produced by the rheumatoid synovium. Arthritis Rheum 42: 963–970. 10323452 10.1002/1529-0131(199905)42:5<963::AID-ANR15>3.0.CO;2-E

[6] Miossec P, Korn T, Kuchroo VK (2009) Interleukin-17 and type 17 helper T cells. N Engl J Med 361: 888–898. 19710487 10.1056/NEJMra0707449

[7] Tartour E, Fossiez F, Joyeux I, Galinha A, Gey A, et al (1999) Interleukin 17, a T-cell-derived cytokine, promotes tumorigenicity of human cervical tumors in nude mice. Cancer Res 59: 3698–3704. 10446984

[8] Miyahara Y, Odunsi K, Chen W, Peng G, Matsuzaki J, et al (2008) Generation and regulation of human CD4+ IL-17-producing T cells in ovarian cancer. Proc Natl Acad Sci U S A 105: 15505–15510. 18832156 10.1073/pnas.0710686105 PMC2563129

[9] Sfanos KS, Bruno TC, Maris CH, Xu L, Thoburn CJ, et al (2008) Phenotypic analysis of prostate-infiltrating lymphocytes reveals TH17 and Treg skewing. Clin Cancer Res 14: 3254–3261. 18519750 10.1158/1078-0432.CCR-07-5164 PMC3082357

[10] Le Gouvello S, Bastuji-Garin S, Aloulou N, Mansour H, Chaumette MT, et al (2008) High prevalence of Foxp3 and IL17 in MMR-proficient colorectal carcinomas. Gut 57: 772–779. 17965063 10.1136/gut.2007.123794

[11] Kirshberg S, Izhar U, Amir G, Demma J, Vernea F, et al (2011) Involvement of CCR6/CCL20/IL-17 axis in NSCLC disease progression. PLoS One 6: e24856. 21949768 10.1371/journal.pone.0024856 PMC3174223

[12] Kuang DM, Peng C, Zhao Q, Wu Y, Chen MS, et al (2010) Activated monocytes in peritumoral stroma of hepatocellular carcinoma promote expansion of memory T helper 17 cells. Hepatology 51: 154–164. 19902483 10.1002/hep.23291

[13] Zhang B, Rong G, Wei H, Zhang M, Bi J, et al (2008) The prevalence of Th17 cells in patients with gastric cancer. Biochem Biophys Res Commun 374: 533–537. 18655770 10.1016/j.bbrc.2008.07.060

[14] Lv L, Pan K, Li XD, She KL, Zhao JJ, et al (2011) The Accumulation and Prognosis Value of Tumor Infiltrating IL-17 Producing Cells in Esophageal Squamous Cell Carcinoma. PLoS One 6: e18219. 21483813 10.1371/journal.pone.0018219 PMC3069054

[15] Kryczek I, Banerjee M, Cheng P, Vatan L, Szeliga W, et al (2009) Phenotype, distribution, generation, and functional and clinical relevance of Th17 cells in the human tumor environments. Blood 114: 1141–1149. 19470694 10.1182/blood-2009-03-208249 PMC2723011

[16] Numasaki M, Fukushi J, Ono M, Narula SK, Zavodny PJ, et al (2003) Interleukin-17 promotes angiogenesis and tumor growth. Blood 101: 2620–2627. 12411307 10.1182/blood-2002-05-1461

[17] Numasaki M, Watanabe M, Suzuki T, Takahashi H, Nakamura A, et al (2005) IL-17 enhances the net angiogenic activity and in vivo growth of human non-small cell lung cancer in SCID mice through promoting CXCR-2-dependent angiogenesis. J Immunol 175: 6177–6189. 16237115 10.4049/jimmunol.175.9.6177

[18] Hirahara N, Nio Y, Sasaki S, Minari Y, Takamura M, et al (2001) Inoculation of human interleukin-17 gene-transfected Meth-A fibrosarcoma cells induces T cell-dependent tumor-specific immunity in mice. Oncology 61: 79–89. 11474253 10.1159/000055357

[19] Benchetrit F, Ciree A, Vives V, Warnier G, Gey A, et al (2002) Interleukin-17 inhibits tumor cell growth by means of a T-cell-dependent mechanism. Blood 99: 2114–2121. 11877287 10.1182/blood.v99.6.2114

[20] Wang L, Yi T, Kortylewski M, Pardoll DM, Zeng D, et al (2009) IL-17 can promote tumor growth through an IL-6-Stat3 signaling pathway. J Exp Med 206: 1457–1464. 19564351 10.1084/jem.20090207 PMC2715087

[21] Kryczek I, Wei S, Szeliga W, Vatan L, Zou W (2009) Endogenous IL-17 contributes to reduced tumor growth and metastasis. Blood 114: 357–359. 19289853 10.1182/blood-2008-09-177360 PMC2714210

[22] Ojima T, Iwahashi M, Nakamura M, Matsuda K, Nakamori M, et al (2007) Successful cancer vaccine therapy for carcinoembryonic antigen (CEA)-expressing colon cancer using genetically modified dendritic cells that express CEA and T helper-type 1 cytokines in CEA transgenic mice. Int J Cancer 120: 585–593. 17096339 10.1002/ijc.22298

[23] Yu P, Lee Y, Liu W, Krausz T, Chong A, et al (2005) Intratumor depletion of CD4+ cells unmasks tumor immunogenicity leading to the rejection of late-stage tumors. J Exp Med 201: 779–791. 15753211 10.1084/jem.20041684 PMC2212829

[24] Borovikova LV, Ivanova S, Zhang M, Yang H, Botchkina GI, et al (2000) Vagus nerve stimulation attenuates the systemic inflammatory response to endotoxin. Nature 405: 458–462. 10839541 10.1038/35013070

[25] Jablonska J, Leschner S, Westphal K, Lienenklaus S, Weiss S (2010) Neutrophils responsive to endogenous IFN-beta regulate tumor angiogenesis and growth in a mouse tumor model. J Clin Invest 120: 1151–1164. 20237412 10.1172/JCI37223 PMC2846036

[26] Weidner N, Semple JP, Welch WR, Folkman J (1991) Tumor angiogenesis and metastasis–correlation in invasive breast carcinoma. N Engl J Med 324: 1–8. 10.1056/NEJM199101033240101 1701519

[27] Bergers G, Brekken R, McMahon G, Vu TH, Itoh T, et al (2000) Matrix metalloproteinase-9 triggers the angiogenic switch during carcinogenesis. Nat Cell Biol 2: 737–744. 11025665 10.1038/35036374 PMC2852586

[28] Ostrand-Rosenberg S, Sinha P (2009) Myeloid-derived suppressor cells: linking inflammation and cancer. J Immunol 182: 4499–4506. 19342621 10.4049/jimmunol.0802740 PMC2810498

[29] Gabrilovich DI, Nagaraj S (2009) Myeloid-derived suppressor cells as regulators of the immune system. Nat Rev Immunol 9: 162–174. 19197294 10.1038/nri2506 PMC2828349

[30] Wu S, Rhee KJ, Albesiano E, Rabizadeh S, Wu X, et al (2009) A human colonic commensal promotes colon tumorigenesis via activation of T helper type 17 T cell responses. Nat Med 15: 1016–1022. 19701202 10.1038/nm.2015 PMC3034219

[31] Xiao M, Wang C, Zhang J, Li Z, Zhao X, et al (2009) IFNgamma promotes papilloma development by up-regulating Th17-associated inflammation. Cancer Res 69: 2010–2017. 19244111 10.1158/0008-5472.CAN-08-3479

[32] Wakita D, Sumida K, Iwakura Y, Nishikawa H, Ohkuri T, et al (2010) Tumor-infiltrating IL-17-producing gammadelta T cells support the progression of tumor by promoting angiogenesis. Eur J Immunol 40: 1927–1937. 20397212 10.1002/eji.200940157

[33] He D, Li H, Yusuf N, Elmets CA, Li J, et al (2010) IL-17 Promotes Tumor Development through the Induction of Tumor Promoting Microenvironments at Tumor Sites and Myeloid-Derived Suppressor Cells. J Immunol 184: 2281–2288. 20118280 10.4049/jimmunol.0902574 PMC3179912

[34] Zhao Q, Xiao X, Wu Y, Wei Y, Zhu LY, et al (2011) Interleukin-17-educated monocytes suppress cytotoxic T-cell function through B7-H1 in hepatocellular carcinoma patients. Eur J Immunol 41: 2314–2322. 21674477 10.1002/eji.201041282

[35] Awasthi A, Kuchroo VK (2009) IL-17A directly inhibits TH1 cells and thereby suppresses development of intestinal inflammation. Nat Immunol 10: 568–570. 19448657 10.1038/ni0609-568

[36] O'Connor W Jr, Kamanaka M, Booth CJ, Town T, Nakae S, et al (2009) A protective function for interleukin 17A in T cell-mediated intestinal inflammation. Nat Immunol 10: 603–609. 19448631 10.1038/ni.1736 PMC2709990

[37] Reppert S, Boross I, Koslowski M, Tureci O, Koch S, et al (2011) A role for T-bet-mediated tumour immune surveillance in anti-IL-17A treatment of lung cancer. Nat Commun 2: 600. 22186896 10.1038/ncomms1609

[38] Martin-Orozco N, Muranski P, Chung Y, Yang XO, Yamazaki T, et al (2009) T helper 17 cells promote cytotoxic T cell activation in tumor immunity. Immunity 31: 787–798. 19879162 10.1016/j.immuni.2009.09.014 PMC2787786

[39] Muranski P, Boni A, Antony PA, Cassard L, Irvine KR, et al (2008) Tumor-specific Th17-polarized cells eradicate large established melanoma. Blood 112: 362–373. 18354038 10.1182/blood-2007-11-120998 PMC2442746

[40] Murdoch C, Muthana M, Coffelt SB, Lewis CE (2008) The role of myeloid cells in the promotion of tumour angiogenesis. Nat Rev Cancer 8: 618–631. 18633355 10.1038/nrc2444

[41] Shahrara S, Pickens SR, Mandelin AM 2nd, Karpus WJ, Huang Q, et al (2010) IL-17-mediated monocyte migration occurs partially through CC chemokine ligand 2/monocyte chemoattractant protein-1 induction. J Immunol 184: 4479–4487. 20228199 10.4049/jimmunol.0901942 PMC2858914

[42] Murugaiyan G, Saha B (2009) Protumor vs antitumor functions of IL-17. J Immunol 183: 4169–4175. 19767566 10.4049/jimmunol.0901017

[43] Ogura H, Murakami M, Okuyama Y, Tsuruoka M, Kitabayashi C, et al (2008) Interleukin-17 promotes autoimmunity by triggering a positive-feedback loop via interleukin-6 induction. Immunity 29: 628–636. 18848474 10.1016/j.immuni.2008.07.018

[44] Miyamoto M, Prause O, Sjostrand M, Laan M, Lotvall J, et al (2003) Endogenous IL-17 as a mediator of neutrophil recruitment caused by endotoxin exposure in mouse airways. J Immunol 170: 4665–4672. 12707345 10.4049/jimmunol.170.9.4665

[45] Charles KA, Kulbe H, Soper R, Escorcio-Correia M, Lawrence T, et al (2009) The tumor-promoting actions of TNF-alpha involve TNFR1 and IL-17 in ovarian cancer in mice and humans. J Clin Invest 119: 3011–3023. 19741298 10.1172/JCI39065 PMC2752076

[46] Wang L, Yi T, Zhang W, Pardoll DM, Yu H (2010) IL-17 enhances tumor development in carcinogen-induced skin cancer. Cancer Res 70: 10112–10120. 21159633 10.1158/0008-5472.CAN-10-0775 PMC3059780

[47] Zhu J, Paul WE (2010) Peripheral CD4+ T-cell differentiation regulated by networks of cytokines and transcription factors. Immunol Rev 238: 247–262. 20969597 10.1111/j.1600-065X.2010.00951.x PMC2975272

[48] Kryczek I, Wei S, Zou L, Altuwaijri S, Szeliga W, et al (2007) Cutting edge: Th17 and regulatory T cell dynamics and the regulation by IL-2 in the tumor microenvironment. J Immunol 178: 6730–6733. 17513719 10.4049/jimmunol.178.11.6730

[49] Yang Z, Zhang B, Li D, Lv M, Huang C, et al (2010) Mast cells mobilize myeloid-derived suppressor cells and Treg cells in tumor microenvironment via IL-17 pathway in murine hepatocarcinoma model. PLoS One 5: e8922. 20111717 10.1371/journal.pone.0008922 PMC2811741

